# Comparative Transcriptome Analysis Reveals bmo-miR-6497-3p Regulate Circadian Clock Genes during the Embryonic Diapause Induction Process in Bivoltine Silkworm

**DOI:** 10.3390/insects12080739

**Published:** 2021-08-18

**Authors:** Lulu Liu, Pan Zhang, Qiang Gao, Xiaoge Feng, Lan Han, Fengbin Zhang, Yanmin Bai, Minjin Han, Hai Hu, Fangyin Dai, Gaojun Zhang, Xiaoling Tong

**Affiliations:** 1 State Key Laboratory of Silkworm Genome Biology, Key Laboratory of Sericultural Biology and Genetic Breeding, Ministry of Agriculture and Rural Affairs, College of Sericulture, Textile and Biomass Sciences, Southwest University, Chongqing 400715, China; liululuswu@163.com (L.L.); gq620520@email.swu.edu.cn (Q.G.); baixiaolailai@163.com (Y.B.); minjinhan@126.com (M.H.); huhaiswu@163.com (H.H.); fydai@swu.edu.cn (F.D.); 2 College of Sericulture, Textile and Biomass Sciences, Southwest University, Chongqing 400715, China; zhangpanpan@email.swu.edu.cn (P.Z.); f19936092062@163.com (X.F.); hl820179802@email.swu.edu.cn (L.H.); fengbin@163.com (F.Z.)

**Keywords:** circadian clock, diapause induction, microRNA, silkworm, temperature, polyphenism, epigenetic regulation

## Abstract

**Simple Summary:**

Diapause is a type of seasonal polyphenism and is induced by multiple cues, such as light and temperature. However, the molecular mechanisms of how the environment induces diapause remains unclear. We are interested in the epigenetic regulation, especially the non-coding RNA, involved in environmentally induced diapause. The progeny eggs of bivoltine silkworm strains undergoing diapause or not depends on the incubation temperature during the maternal embryonic stage. In this research, we compared the differentially expressed microRNAs in embryos incubated under diapause-inducing temperature (25 °C) and non-diapause-inducing temperature (15 °C) in silkworm. Our results indicate that miRNAs respond to diapause-inducing temperature and regulate the expression of circadian clock genes. Our research not only provides reference for the study of the diapause induction process, but also gives insights into the role of epigenetic modification in diapause.

**Abstract:**

Diapause is one of the survival strategies of insects for confronting adverse environmental conditions. *Bombyx mori* displays typical embryonic diapause, and offspring diapause depends on the incubation environment of the maternal embryo in the bivoltine strains of the silkworm. However, the molecular mechanisms of the diapause induction process are still poorly understood. In this study, we compared the differentially expressed miRNAs (DEmiRs) in bivoltine silkworm embryos incubated at diapause- (25 °C) and non-diapause (15 °C)-inducing temperatures during the blastokinesis (BK) and head pigmentation (HP) phases using transcriptome sequencing. There were 411 known miRNAs and 71 novel miRNAs identified during the two phases. Among those miRNAs, there were 108 and 74 DEmiRs in the BK and HP groups, respectively. By the Kyoto Encyclopedia of Genes and Genomes (KEGG) analysis of the predicted target genes of the DEmiRs, we found that aside from metabolism, the targets were also enriched in phototransduction-fly and insect hormone biosynthesis in the BK group and the HP group, respectively. Dual luciferase reporter assay illustrated that bmo-miR-6497-3p directly regulated *Bmcycle* and subsequently regulated the expression of circadian genes. These results imply that microRNAs, as vitally important regulators, respond to different temperatures and participate in the diapause induction process across species.

## 1. Introduction

Diapause is a crucial survival strategy by which insects confront adverse environmental conditions [1], and the timing of diapause is beneficial to synchronizing populations [2]. Insect diapause can be divided into four types according to the different stages of occurrence: embryonic diapause, larval diapause, pupal diapause, and adult diapause. Several environmental factors could induce diapause, such as photoperiod, temperature, humidity, food availability, and population density [3]. The biological clock is altered by external environmental changes and the signal is sent through endocrine hormones to the downstream genes, causing organism biochemical and physiological changes, which result in diapause [4]. Ecdysone and prothoracicotropic hormone (PTTH) play important roles in the initiation, maintenance, and termination of pupal diapause [5,6,7], while juvenile hormone (JH) is responsible for larval diapause and adult diapause [8,9,10]. However, the molecular mechanism of diapause induction is not well understood.

Diapause, as a phenomenon of seasonal rhythms, can be induced by the zeitgebers of temperature and photoperiod that affect the circadian clock [11,12,13,14]. The circadian clock system is composed of three parts: the input pathway, the central oscillator, and the output pathway [15,16,17,18]. Diapause can be induced when the temperature and photoperiod zeitgebers that act as the external environment are input to the circadian clock system to reset the endogenous biological clock, and are translated into relevant hormonal signals. The temperature and photoperiod zeitgebers in the external environment are input to the circadian clock system; entrainment resets the endogenous biological clock, resulting in relevant hormonal signals that eventually induce insect diapause [19]. There is an abundance of evidence that proves the circadian clock is closely related to insect diapause. In *Drosophila melanogaster*, lack of the *timeless* gene leads to a reduction in diapause rates [15]. Similarly, there are several studies which reported that non-diapause-destined female mosquitoes *Culex pipiens* [20] entered diapause after RNA interference with two circadian clock positive feedback regulation genes, while diapause-destined female mosquitoes continued to develop directly after interfering with negative feedback regulation genes. In *Riptortus pedestris* [21,22], circadian clock genes participate in adult diapause by regulating the secretion of JH. To date, the molecular mechanisms underlying how photoperiod affects internal circadian rhythms have been clarified in detail in multiple species [23,24,25,26], but how the environmental temperature regulates internal circadian rhythms and participates in the diapause induction process is unknown.

The phenomenon that organisms could exhibit phenotypic plasticity under different environmental conditions is called polyphenism. Diapause of the bivoltine silkworm is a typical seasonal polyphenism, and it has attracted much attention in the field of biology. The current research on polyphenism is mainly on the variation in epigenetic factors, including DNA methylation, non-coding RNA, and histone modification, which are involved in the regulation of expression of genes under different abiotic stresses. MicroRNAs (miRNAs), a type of endogenous non-coding RNA about 18–25 nt in length, could bind to the seed sequence site of 3′UTR of target genes to silence the genes via degrading transcripts or inhibiting translation [27,28,29,30]. Previous studies have shown that the expression profiles of miRNAs as a kind of epigenetic modification may change in response to environmental stressors such as oxygen deprivation [31], dehydration [32,33], freezing [34,35], and starvation [36]. In addition, miRNAs were also reported to participate in metabolism apart from diapause-relevant physiological processes such as [37,38] cell-cycle progression [39,40], stress resistance [41,42], and developmental timing [43,44]. Additionally, miRNAs also regulate diapause initiation, maintenance, and termination [45,46]. For example, changes in the miRNA expression profile are closely related to adult diapause in the mosquito, *Culex pipiens* [47], and to pupal diapause in the flesh fly, *Sarcophaga bullata* [48]. The accumulated evidence suggests the possibility that miRNAs may respond to temperature variation and subsequently regulate the induction of insect diapause.

To investigate the role of miRNAs in the process of environmental temperature changes affecting internal circadian rhythms to influence progeny diapause, we focused on those miRNAs that respond to temperature and that target circadian clock genes. The bivoltine silkworm is an ideal species for this research, as *Bombyx mori* is a typical embryonic diapause insect, and the diapause of bivoltine silkworms is mainly determined by environmental signals, especially the temperature perceived by the embryo during the temperature-sensing window (stages 20–23) [49]. Female adult silkworms grown from eggs incubated at 25 °C under continuous light will produce diapause eggs, while female silkworms grown from eggs incubated at 15 °C in continuous darkness will produce non-diapause eggs [50,51]. In this study, next-generation sequencing was utilized to compare the differentially expressed miRNAs in different temperature-treated embryos during the blastokinesis (BK) phase and head pigmentation (HP) phase in DaZao, a bivoltine strain of the silkworm. The BK phase (embryo development 4–5 days) represents stage 21, which belongs to the temperature-sensing window, so the groups of BK25 and BK15 were compared to explore the miRNAs involved in responding to temperature during the diapause-sensitive period. The environmental signals sensed during the BK stage would be converted into the endocrine hormone signals to induce diapause in the later stage of embryonic development. The HP phase (embryo development 6–7 days) belongs to the late development phase in the embryonic period; thus, the groups of HP25 and HP15 were used to identify the miRNAs participating in storing the diapause-related signal. Our research provides a reference for the role of miRNAs participating in the regulation of circadian rhythm during the diapause induction process.

## 2. Materials and Methods

### 2.1. Materials

DaZao, a bivoltine strain of the silkworm, was obtained from the Silkworm Gene Bank of Southwest University, Chongqing, China. The non-diapause eggs were divided into two groups; one group was incubated at 25 °C (diapause induction temperature), while the other group was incubated at 15 °C (non-diapause induction temperature). All the eggs were incubated at the same light condition (continuous darkness). Four groups were prepared from embryos exposed to the two temperature treatments (25 and 15 °C) and in the two phases (the blastokinesis (BK) and head pigmentation (HP) phases). Due to the non-uniform development of embryos, we dissected a great deal of the silkworm eggs and selected the embryos showing the same morphology at the same developmental stage for subsequent sequencing. The resulting four groups were labeled BK25, BK15, HP25, and HP15. Each group comprised 100 embryos. All samples were stored at −80 °C for further use. The cell line, BmE, derived from the silkworm embryo, was cultured at 27 °C in Grace insect medium (Life Technologies, Shanghai, China) supplemented with 10% fetal bovine serum (United States Biological, Swampscott, MA, USA), penicillin (200 U/mL), and streptomycin (200 U/mL).

### 2.2. mRNA and sRNA Library Construction and Sequencing

Total RNA of all samples was isolated using TRIzol (Invitrogen, Carlsbad, CA, USA) following the manufacturer’s protocol. RNA degradation was detected on 1% agarose gels. RNA contamination was evaluated by the ratio of OD260/OD280 and OD260/OD230. RNA purity was checked using the NanoPhotometer^®^ spectrophotometer (IMPLEN, Los Angeles, CA, USA). RNA concentration was measured using a Qubit^®^ RNA Assay Kit with a Qubit^®^ 2.0 Fluorimeter (Life Technologies, Carlsbad, CA, USA). RNA integrity was assessed using the RNA Nano 6000 Assay Kit of the Agilent Bioanalyzer 2100 system (Agilent Technologies, Santa Clara, CA, USA). A total of 6 μg total RNA per sample was used as input material, respectively, for the mRNA and small RNA library. Sequencing libraries were generated using the rRNA depleted RNA by NEBNext^®^ Ultra™ Directional RNA Library Prep Kit for Illumina^®^ (NEB, Ipswich, MA, USA) and using NEBNext^®^ Multiplex Small RNA Library Prep Set for Illumina^®^ (NEB, Ipswich, MA, USA) following the manufacturer’s recommendations, and index codes were added to attribute sequences for each sample. For mRNA sequencing, in order to select cDNA fragments of preferentially 150~200 bp in length, the library fragments were purified with AMPure XP system (Beckman Coulter, Beverly, CA, USA). Then, 3 μL USER Enzyme (NEB, Ipswich, MA, USA) was used with size-selected, adaptor-ligated cDNA at 37 °C for 15 min followed by 5 min at 95 °C before PCR. Then, PCR was performed with Phusion High-Fidelity DNA polymerase, universal PCR primers, and index (X) primer. Briefly, in sRNA sequencing, NEB 3′ SR adaptor was directly and specifically ligated to the 3′ ends of miRNA, siRNA, and piRNA. After the 3′ ligation reaction, the SR RT primer was hybridized to the excess of 3′ SR adaptor (that remained free after the 3′ ligation reaction), and the single-stranded DNA adaptor was transformed into a double-stranded DNA molecule. This step is important to prevent adaptor dimer formation; in addition, dsDNAs are not substrates for ligation mediated by T4 RNA ligase 1 and therefore do not ligate to the 5′ SR adaptor in the subsequent ligation step. The 5′ end adaptor was ligated to the 5′ ends of miRNAs, siRNA, and piRNA. Then, first-strand cDNA was synthesized using M-MuLV Reverse Transcriptase (RNase H–). PCR amplification was performed using LongAmp Taq 2X Master Mix, SR primer for Illumina, and index (X) primer. PCR products were purified on an 8% polyacrylamide gel (100 V, 80 min). DNA fragments corresponding to 140–160 bp (the length of small noncoding RNAs plus the 3′ and 5′ adaptors) were recovered and dissolved in 8 μL elution buffer. Finally, the quality of all libraries was assessed on an Agilent Bioanalyzer 2100 system using DNA High Sensitivity Chips.

Raw data (raw reads) in fastq format were first processed through custom perl and python scripts. In this step, clean data (clean reads) were obtained by removing reads containing poly-N, those with 5′ adaptor contaminants, those without the 3′ adaptor or the insert tag, those containing poly-A, -T, -G, or -C, and low-quality reads from the raw data. At the same time, Q20, Q30, and GC content of the raw data were calculated. Then, we chose a certain range of lengths from the clean reads to perform the downstream analyses.

### 2.3. miRNA Identification

The small RNA tags were mapped to the reference sequence by Bowtie without mismatch to analyze their expression and distribution on the reference sequence [52]. Mapped small RNA tags were used to search for known miRNAs. The program miRBase20.0 was used as a reference, and modified software programs mirdeep2 [53] and srna-tools-cli were used to obtain the potential miRNAs and to draw their secondary structures. The characteristic hairpin structure of miRNA precursors was used to predict novel miRNAs. The available software miREvo and mirdeep2 were integrated to predict novel miRNAs through exploring the secondary structure, the Dicer cleavage site, and the minimum free energy of the small RNA tags unannotated in the preceding steps [53,54]. In our analysis pipeline, known miRNAs used miFam.dat (http://www.mirbase.org/ftp.shtml (last accessed on 14 October 2019)) to search for families; novel miRNA precursors were submitted to Rfam (http://rfam.sanger.ac.uk/search/ (last accessed on 9 May 2018)) to search for Rfam families.

### 2.4. Screening of Differentially Expressed miRNAs

The miRNA expression levels were estimated by TPM (transcripts per million) through the following criteria [55]: Normalization formula: Normalized expression = mapped read count/Total reads × 10^6^. Differential expression analysis of two samples was performed using the DEGseq (2010) R package. *p*-values were adjusted using q values [56]. A q value < 0.01 and |log2(foldchange)| > 1 were set as the default thresholds for significantly differential expression.

### 2.5. GO and KEGG Enrichment Analysis of Predicted Target Genes of the DEmiRs

To investigate the biological processes that DEmiRs were involved in under diapause- and non-diapause-inducing temperatures during the BK and HP phases, we further performed Gene Ontology (GO) annotation and Kyoto Encyclopedia of Genes and Genomes (KEGG) enrichment analysis on the differentially expressed candidate target (DECT) genes of the DEmiRs in the BK and HP groups.

### 2.6. Construction of miRNA Overexpression Vectors

To construct an miRNA overexpression vector, we first determined the position of the pre-miRNA on the genome, then extended 200 bp upstream and downstream of thispre-miRNA, respectively, to obtain the sequence of the target fragment, which was then constructed into the PIZ vector. Finally, we evaluated the efficiency of miRNA overexpression vectors in silkworm embryo cells.

### 2.7. Quantitative Real-Time PCR (qRT-PCR)

Total RNA of different temperature-treated embryos at the BK phase and HP phase was extracted with an E.Z.N.A.^®^ Total RNA Kit I (Omega Bio-Tek, Guang Zhou, China) according to the manufacturer’s instructions. A PrimeScript™ RT reagent kit with gDNA Eraser (Perfect Real Time) was applied to synthesize first-strand cDNA. Random Primer p(dN)6 was used to construct mRNA cDNA, and Stem loop (SL) primers were used to synthesize miRNA cDNA (Table S2). All of the primers were synthesized by Tsingke (Chongqing, China). The first-strand and specific cDNAs were used as templates in the amplification of cycle and bmo-miR-6497-3p. Briefly, the PCR reaction procedure was performed as follows: 10 μL of Hieff^®^ qPCR SYBR Green Master Mix (No Rox), 0.4 μL of 10 μM primer F, 0.4 μL of 10 μM primer R, 8.2 μL of ddH2O, and 1 μL of cDNA template were mixed at 95 °C for 5 min, followed by 40 cycles at 95 °C for 10 s and 60 °C for 30 s. *TIF-4A* (BGIBMGA003186) and U6 were used as internal control genes for detecting the expression levels of cycle and bmo-miR-6497-3p, respectively [57]. Additionally, the relative expression ratio of the target genes was analyzed using the 2^−ΔΔCt^ method [58].

### 2.8. Dual Luciferase Reporter (DLR) Assay

The dual luciferase expression vector and the Renilla vector were purchased from Thermo Fisher Scientific, Waltham, MA, USA. *Bmcycle* and *Bmpdp* 3′UTR (400 bp), which contained approximately 200 bp upstream and downstream of the miRNA binding site, were cloned and inserted downstream of the ORF of firefly luciferase. The BmE cell line was used for the DLR assay. Cells were plated in a 12-well plate. Transfection was performed at 80% confluence with X-tremeGENE™ HP DNA transfection reagent (Roche, Mannheim, Germany) according to the manufacturer’s instructions. In the DLR assay, 500 ug of miRNA expression plasmid or empty vector, 500 ng of the firefly luciferase reporter or control firefly luciferase vector, and 100 ng of Renilla Luciferase vector were co-transfected in each cell well. Luciferase assays (Dual Luciferase System, Promega, Madison, WI, USA) were performed 72 h after co-transfection. Renilla luciferase activity provided normalization for firefly luciferase activity.

## 3. Results

### 3.1. Quality Control and miRNA Identification

**Figure 1:**
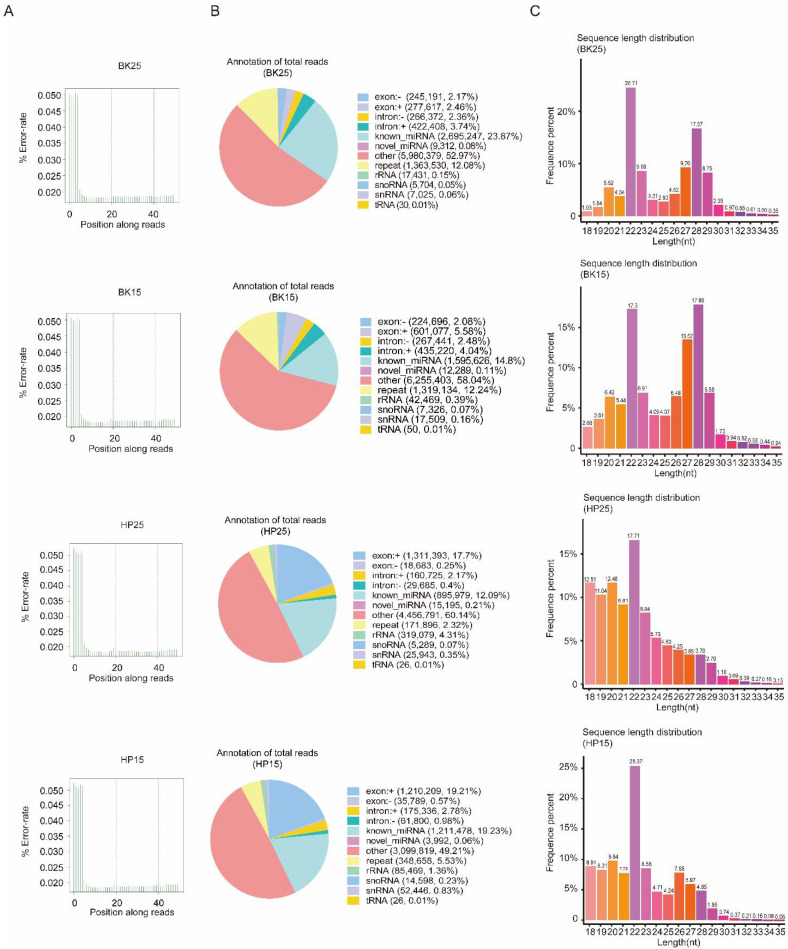
Quality control of sequencing data and distribution of non-coding RNA (ncRNA). (**A**) The distribution of sequencing error rate in BK25, BK15, HP25, and HP15. Owing to the incomplete match between the random primer and the RNA template, the sequencing error rate of the first few bases reached about 0.05. For convenience, “BK25” and “BK15” represent the embryos incubated at 25 and 15 °C during the blastokinesis phase, respectively; “HP25” and “HP15” represent the embryos incubated at 25 and 15 °C, respectively, during the head pigmentation phase. (**B**) Distribution of ncRNA in BK25, BK15, HP25, and HP15. (**C**) Length distribution of sRNA in BK25, BK15, HP25, and HP15. nt, nucleotide.

There were 13,743,258 clean reads in the BK25 group, 14,511,849 clean reads in the BK15 group, 13,803,264 clean reads in the HP25 group, and 13,787,630 clean reads in the HP15 group in small RNA sequencing libraries. Among the clean reads, 11,290,246, 10,778,240, 7,410,664, and 6,299,620 were mapped to the chromosomes of *Bombyx mori* (Table S1). The quality control results indicate that there was no presence of sequencing errors at the base position with a rate above 0.05%, and the sequence results were deemed credible for the subsequent analysis (Figure 1A). The proportion of miRNAs containing novel and known miRNAs in total reads were 23.95%, 14.91%, 12.3%, and 19.29% in the BK25 group, BK15 group, HP25 group, and HP15 group, respectively, and there were 0.27%, 0.53%, 4.74%, and 2.43% identified as other sRNAs, containing ribosome RNAs (rRNA), small nucleolar RNA (snoRNA), small nuclear RNA (snRNA), and transfer RNA (tRNA). In total, there were 481 miRNAs, and 411 known miRNAs and 70 new miRNAs were identified in the transcriptome data by miREvo and mirdeep2. The sequence length distribution of sRNA showed that the length distribution of miRNA ranged from 20 to 23 nt, mostly 22 nt in the BK25 and BK15 groups, while the length distribution of miRNA in the HP25 and HP15 groups ranged from 18 to 23 nt, mostly 22 nt (Figure 1C).

### 3.2. Differential Expression Analysis of miRNAs

**Figure 2:**
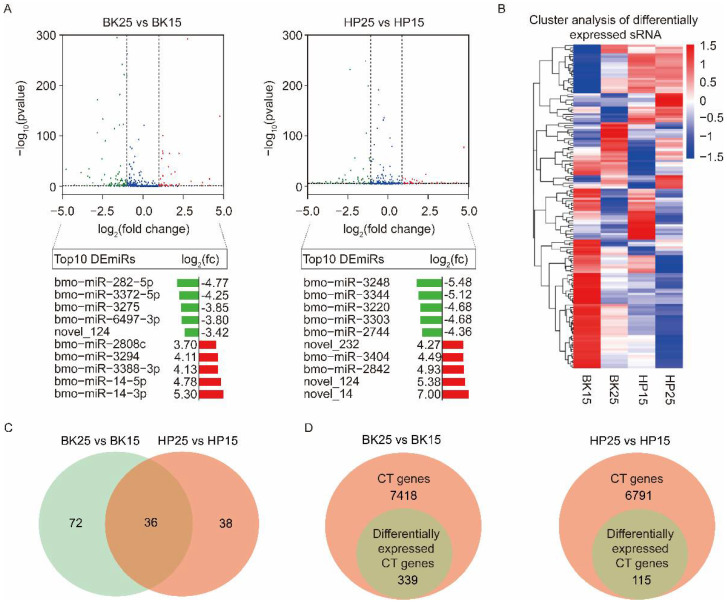
Integrated analyses of differentially expressed miRNA (DEmiRs) and identification of candidate genes of DEmiRs. (**A**) Volcano plots of DEmiRs at different temperatures during the BK and HP phases. The blue plots represent those miRNAs that were not significantly differentially expressed at different temperatures. The green plots represent those miRNAs that were downregulated at 25 °C compared with 15 °C during the BK and HP phases; the red plots represent those miRNAs that were upregulated at 25 °C compared with 15 °C during the BK and HP phases. The log2(fc) of the top 10 upregulated and downregulated DEmiRs at 25 °C compared with 15 °C during the BK and HP phases are shown under the corresponding volcano plots. (**B**) Heat map representing the expression levels of DEmiRs under different temperatures during the BK and HP phases. (**C**) Venn diagram representing the DEmiRs under different temperatures during both BK and HP phases. (**D**) Venn diagram representing the candidate target (CT) genes of DEmiRs that were predicted by miranda, PITA, and RNAhybrid and that were also differentially expressed under different temperatures during the BK and HP phases.

Transcriptome analysis using a comparison of BK25 versus BK15 showed that there were 108 DEmiRs, containing 93 known miRNAs and 15 novel miRNAs. Among the DEmiRs, there were 40 upregulated miRNAs and 68 downregulated miRNAs in the BK25 group compared with the BK15 group. There were 74 DEmiRs containing 62 known miRNAs and 12 novel miRNAs during the HP phase. Among those DEmiRs, there were 27 upregulated miRNAs and 47 downregulated miRNAs in the HP25 group compared with the HP15 group. The expression level changes in the top 10 DEmiRs under different temperatures during the BK and HP phases are shown below the corresponding groups in the volcano map (Figure 2A). The expression profiles of all DEmiRs also are shown in the heat map (Figure 2B). There were 36 miRNAs significantly differentially expressed during the BK and HP phases (Figure 2C), and half of the members of the bmo-miR-2733 family were significantly differentially expressed during both BK and HP phases.

### 3.3. The Identification of Potential Target Genes of DEmiRs

To analyze which DEmiRs might be associated with the process of bivoltine silkworm embryonic diapause induction, we identified the predicted candidate target (CT) genes of miRNAs by examining the intersection of miRanda, PITA, and RNAhybrid software prediction results [59]. There were 7418 and 6791 genes predicted as CT genes of all DEmiRs during the BK and HP phases, respectively. To precisely identify the targets of the DEmiRs, we combined with the mRNA sequencing data and selected the CT genes that showed opposite trends to their corresponding miRNAs and that were significantly differentially expressed in each comparative group under diapause- and non-diapause-inducing temperatures (Figure 2D). The analysis finally identified 339 and 115 genes as differentially expressed candidate target (DECT) genes for the 108 and 74 DEmiRs during the BK and HP phases, respectively (Figure 2D).

### 3.4. Functional Analysis of DECT Genes of DEmiRs

**Figure 3:**
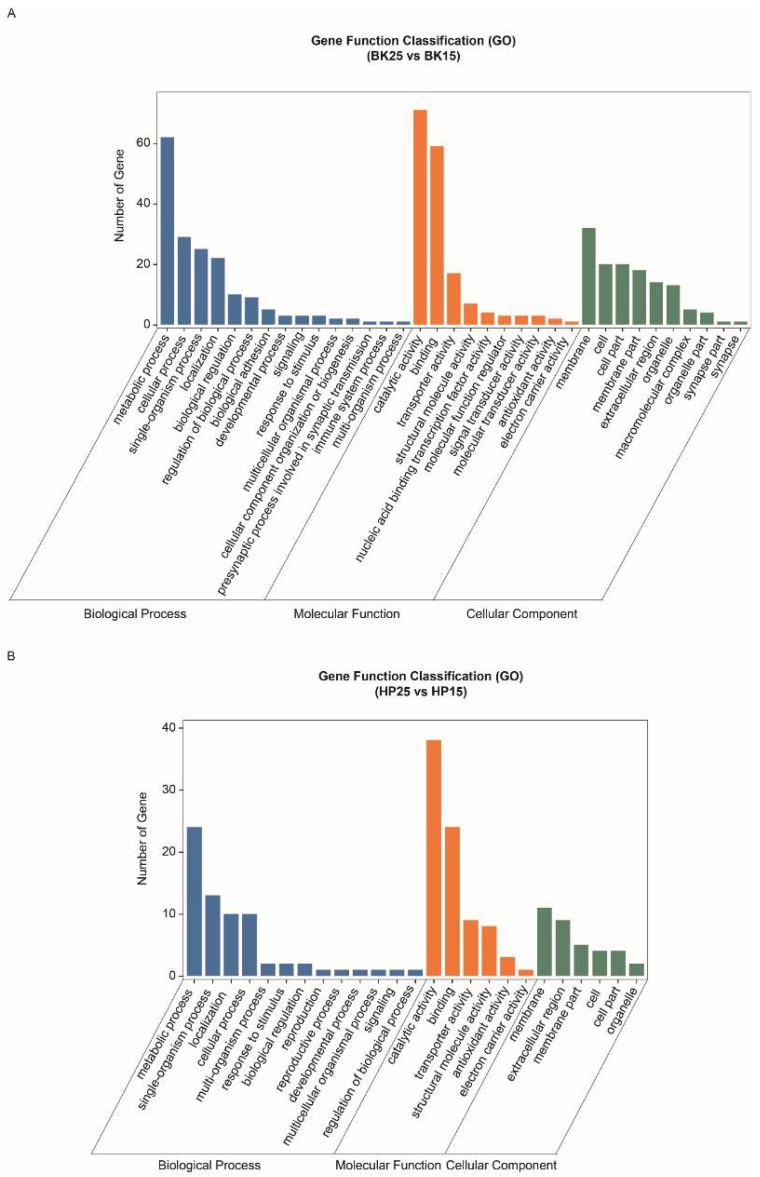
Gene ontology annotation of differentially expressed candidate target (DECT) genes. (**A**) Gene ontology annotation of DECT genes in the BK phase. (**B**) Gene ontology annotation of DECT genes in the HP phase.

**Figure 4:**
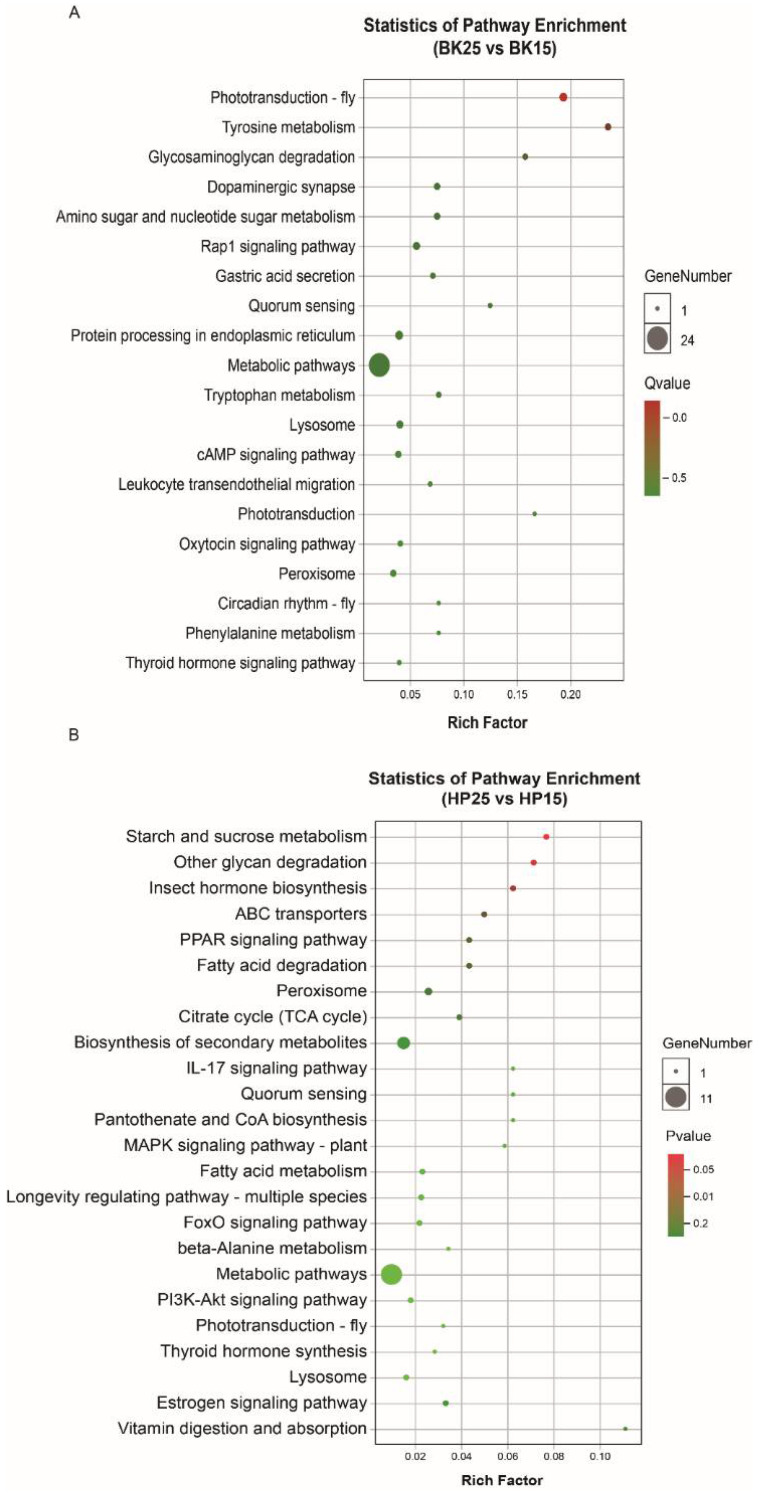
Kyoto Encyclopedia of Genes and Genomes (KEGG) enrichment of DECT genes. (**A**) KEGG pathway enrichment of DECT genes in BK, (**B**) KEGG pathway enrichment of DECT genes in HP. Larger circles indicate more candidate target genes enriched in this pathway, and a q-value/*p*-value closer to 0 represents more significant enrichment.

The GO-annotated enrichment results of DECT genes of DEmiRs during the BK phase show that the DECT genes were mainly related to molecular functions involved in catalytic activity, binding and transporter activity, biological processes including metabolic processes, single-organism processes, cellular processes and localization, and cellular membranes (Figure 3A). Meanwhile, the KEGG enrichment analysis results show that the DECT genes were enriched in a total of 20 pathways (Figure 4A), and the most significantly enriched KEGG pathways were phototransduction-fly and tyrosine metabolism. In addition, most of the DECT genes of DEmiRs participated in metabolic pathways. The circadian rhythm-fly pathway was also enriched under different temperatures during the BK phase.

The GO-annotated enrichment results for DECT genes of DEmiRs during the HP phase show that those genes were mainly related to molecular functions involving catalytic activity and binding, and biological processes, including metabolic processes and single-organism processes (Figure 3B). There were fewer genes involved in the formation of cell membranes. Moreover, the KEGG enrichment analysis results show that these DECT genes were enriched in 24 pathways (Figure 4B). Most of those genes participated in metabolism, and the DECT genes were highly related to the three pathways starch and sucrose metabolism, other glycan degradation, and insect hormone biosynthesis, according to the enrichment factors. Furthermore, other function processes associated with signal transduction pathways such as the FoxO, the MAPK, and the PI3K–Akt signaling pathway also were enriched under diapause- and non-diapause-inducing temperatures during the HP phase.

**Figure 5:**
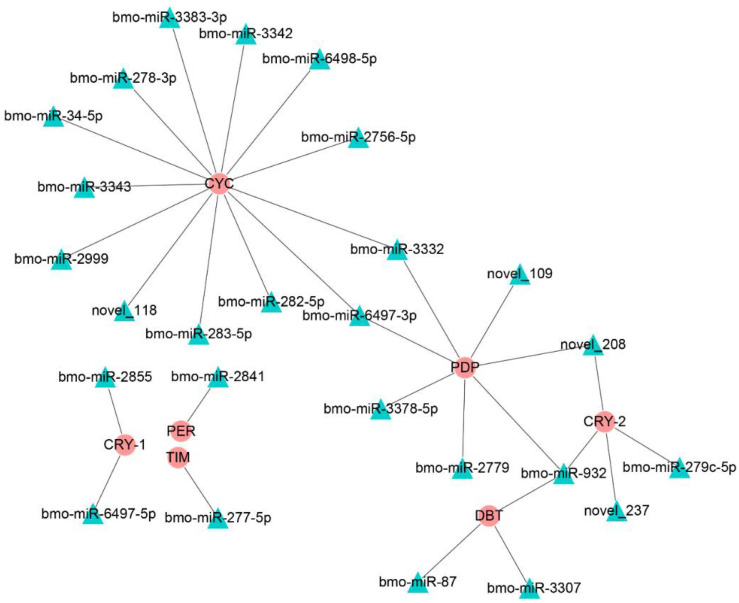
The regulatory networks formed by the Drosophila homologous genes of the circadian clock-fly pathway in the silkworm and differentially expressed miRNAs. The round red nodes represent the circadian clock genes; the green triangular nodes represent differentially expressed miRNAs.

A previous study showed that circadian rhythms were closely related to insect diapause; thus, we focused on the miRNAs that regulated genes in the circadian rhythm-fly pathway (Figure 5). The predicted results show that only the bmo-miR-6497-3p in the top 10 DEmiRs was predicted to regulate *Bmpdp* and *Bmcycle*, the homologs of the Drosophila PAR domain protein gene (*pdp*) and *cycle*, respectively, in the circadian rhythm pathway.

### 3.5. Interaction Analysis of bmo-miR-6497-3p and Circadian Clock Genes

**Figure 6:**
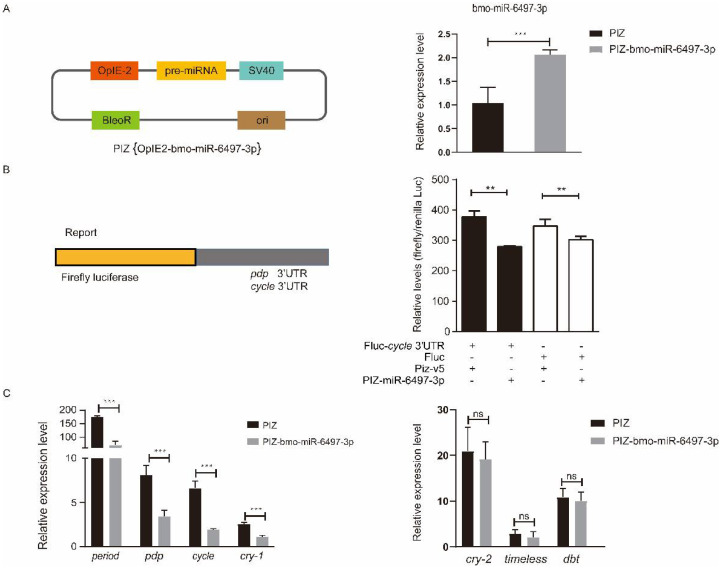
The core circadian clock gene *cycle* is a target of bmo-miR-6497-3p. (**A**) The bmo-miR-6497-3p expression level was significantly upregulated compared with the control group after transfecting the overexpression vector in silkworm embryo cells. (**B**) Luciferase reporter assays were performed after co-transfection with OpIE2-fluc-pdp/cycle 3′UTR or fluc together with an empty vector (PIZ-v5) or a bmo-miR-6497-3p expression vector (PIZ-v5-6497-3p). The Renilla reporter vector was co-transfected with vectors in all groups. For each condition, the luciferase value was normalized by Renilla luciferase and is shown as the mean ± standard deviation (SD). (**C**) bmo-miR-6497-3p inhibits the expression levels of some circadian clock genes in vitro. Silkworm embryo cells transfected with PIZ or PIZ-miR-6497-3p were analyzed for the changes in expression levels of circadian rhythm-related genes. Each experiment was performed three times independently, and all expression profile results obtained with three biological replicates and three technical replicates are shown with means ± SD. “*” represents that 0.01 ≤ *p* value ≤ 0.05, “**” represents that 0.001 ≤ *p* value ≤ 0.01, and “***” represents that *p* value ≤ 0.001.

To explore the relationship between bmo-miR-6497-3p and the circadian clock genes *Bmpdp* and *Bmcycle*, we performed a dual luciferase assay. The vector (PIZ-OpIE2-bmo-miR-6497-3p) was constructed to overexpress bmo-miR-6497-3p in vivo. PGL3-pdp and PGL3-cycle vectors were constructed by ligating the 3′UTR containing the predicted bmo-miR-6497-3p binding sites of the circadian clock genes pdp and cycle into the downstream area of luciferase in the PGL3 vector. We further verified that the expression of bmo-miR-6497-3p was significantly increased in the silkworm embryo cells 72 h after co-transfection (Figure 6A). The luciferase intensity was measured after co-transfecting the PGL3-pdp or PGL3-cycle vectors with bmo-miR-6497-3p overexpressed vector in silkworm embryo cells. The results show that the activity of the luciferase in the PGL3-cycle 3′UTR group was significantly reduced compared to that in the control group at 72 h after co-transfecting with the bmo-miR-6497-3p overexpression vector (Figure 6B). However, there was no significant difference between the PGL3-pdp treatment and the control (data not shown). The results suggest that bmo-miR-6497-3p could directly regulate *Bmcycle* by binding to its 3′UTR. This was further verified by qRT-PCR analysis of the cycle, in which the cycle expression level was significantly reduced after bmo-miR-6497-3p was overexpressed (Figure 6C). Cycle is a central rhythm gene that can regulate the entire circadian clock network by forming a heterodimer with the another one core circadian clock gene *clock*. Hence, we detected the expression levels of the main downstream circadian rhythm genes after transfecting PIZ-miR-6497-3p into BmE cells. The results show that the expression levels of three circadian clock genes (*Bmperiod*, *Bmcry-1*, and *Bmpdp*) were significantly decreased after overexpression of bmo-miR-6497-3p in embryonic cells compared to the control group (Figure 6C).

## 4. Discussion

In the present study, we performed micro-transcriptome sequencing of silkworm embryos incubated under diapause- and non-diapause-inducing temperatures during the BK and HP phases. The BK phase is the critical period during which the silkworm embryo perceives the environment cues, and the HP phase contributes to exploring the miRNAs involved in the transmission of diapause signals [49]. In the micro-transcriptome sequencing results for the BK and HP groups, there were 108 DEmiRs identified, including 15 novel DEmiRs in the BK groups and 74 DEmiRs, including 12 novel DEmiRs, in the HP groups. Among those DEmiRs, we found that the number of upregulated miRNAs was greater than the number of downregulated miRNAs under non-diapause-inducing temperatures compared to that under diapause-inducing temperatures during the BK and HP phases, as illustrated in the heat map of the results. The results coincide with the finding that the number of upregulated DE genes was greater than the number of downregulated genes under diapause-inducing temperatures compared to that under non-diapause-inducing temperatures during the BK and HP phases. The above results imply that miRNAs as the epigenetic regulators may participate in diapause induction by regulating the expression of genes in silkworm embryos under diapause- and non-diapause-inducing temperatures during the BK and HP phases.

Based on the joint analysis of the prediction results of candidate target genes of DEmiRs from the three software programs, 339 and 115 DECT genes were identified as targets for DEmiRs during the BK and HP phases, respectively. To further understand how those DEmiRs functioned and how they are related to the diapause induction process during the BK and HP phases, the enriched GO function and KEGG pathway analyses were performed for those DECT genes under diapause- and non-diapause-inducing temperatures. The DECT genes of all DEmiRs in the BK groups were mainly involved with catalytic activity and binding functions, and most were also related to metabolism processes. In addition, the KEGG enrichment pathways analysis also showed that the DECT genes were involved in metabolism, phototransduction-fly, and tyrosine metabolism pathways. The BK phase is the most vigorous period of organ and tissue formation; therefore, the DECT genes involved in metabolism provide increased levels of energy for the embryo, since the silkworm embryo needs more energy for supporting embryonic development under diapause-inducing temperatures compared to the level needed under non-diapause-inducing temperatures. One of the products of the tyrosine metabolism pathway is dopamine, which plays a vital important role in development in Aedes aegypti [60] and in modulating metabolism and temperature sensitivity in *Drosophila melanogaster* [61,62]. In the present study, the tyrosine metabolism pathways with temperature sensitivity may to some extent be responsible for delaying embryonic development under non-diapause-inducing temperatures during the BK phase. It is worth noting that the phototransduction-fly pathway was also highly enriched under different temperatures during the BK phase. There were genes mainly related to intracellular signal transduction enriched in the phototransduction-fly pathway. This suggested that the temperature signal might share the same signal transduction pathway with the photoperiod signal, and only the macromolecules that receive temperature and light signals differ. Previous studies reported that the transcriptional and translational levels of two circadian clock genes (cryptochrome-2 and period) can respond to temperature changes synchronously in *Bombyx mori* embryos [63]. Thus, we mainly focused on the DEmiRs that potentially target the genes involved in circadian clock pathways or that are related to circadian rhythms. Among those DEmiRs, there were three miRNAs (miR-263a-5p, miR-263b-5p, and bantam-3p) significantly differentially expressed in different temperature-treated embryos during the BK phase, and it was also reported that miR-263a and miR-263b exhibited circadian oscillations and that bantam participates in the regulation of circadian rhythms, even though it does not cycle in Drosophila [64,65]. The most important point is that one of the top 10 miRNAs in the BK groups, bmo-miR-6497-3p, was predicted to regulate *Bmpdp* and *Bmcycle* of the circadian rhythm pathway. The DLR assay and the qRT-PCR results show that the miRNA could regulate *Bmcycle* and eventually modulate the expression of several other circadian clock genes. Previous studies have proved that the circadian clock genes participate in the regulation of diapause in insects [66,67,68]. For example, RNAi targeted against the expression of the positive circadian clock regulators clock and cycle induced non-diapause-destined adults to enter a diapause-like state in *Riptortus pedestris* [21,22,69] and *Pyrrhocoris apterus* [70]. On the contrary, knocking down the expression of the negative circadian regulator period prevented diapause-destined adults from entering the diapause state in *Riptortus pedestris* [22], *Colaphellus bowring* [26], and *Nasonia vitripennis* [71]. Based on the above previous research, we presumed that the circadian clock genes maybe also participate in the regulation of embryonic diapause induction in silkworm. Given that bmo-miR-6497-3p could regulate the expression of several circadian clock genes during the temperature-sensitive period in silkworm, we speculated that bmo-miR-6497-3p might be associated with the diapause induction process in silkworm. However, further knockout experiments are needed to test the hypothesis. Among the top 10 DEmiRs during the BK phase, bmo-miR-3372-5p was only expressed in embryos under non-diapause-inducing temperatures. We found that bmo-miR-3372-5p was mainly involved in transport and catabolism, signal transduction, and sensory system pathways by performing the enriched KEGG pathway analysis of the DECT genes of the miRNA. The downregulation of the genes involved in transport and catabolism is closely related to the lower metabolic rate and slower embryonic development under non-diapause-inducing temperatures during the BK phase. The finding that bmo-miR-3372-5p is also involved in signal transduction and sensory systems suggests that the miRNA might to some extent block the reception and transduction of environmental signals by downregulating the expression of relevant genes. The above lines of evidence suggest that miRNA as a kind of important post-transcriptional regulator could participate in embryonic development, energy metabolism, and regulation of circadian rhythm during environmentally sensitive stages of the bivoltine silkworm embryo under diapause- and non-diapause-inducing temperatures.

The HP phase, the period when embryonic development is nearly complete, is suitable for studying the process of diapause signal transfer. During the HP phase, those DECT genes of all DEmiRs are mainly related to catalytic activity and binding functions and are also relevant to metabolism processes. Moreover, the KEGG enrichment analysis showed that those DECT genes were mainly enriched in metabolism pathways, starch and sucrose metabolism, other glycan degradation, and insect hormone biosynthesis. The DECT genes of all DEmiRs in the first three pathways are involved in providing support for the energy metabolism of silkworm embryonic development under different temperatures during the HP phase. Apart from metabolism, the insect hormone biosynthesis pathway was also enriched under different temperatures during the HP phase. The DECT genes in the pathway were mainly relevant to ecdysone synthesis. Ecdysone as an important hormone is involved in diapause in multiple species. There are reports that the brain can regulate the secretion of ecdysone through the synthesis and release of prothymus hormone (PTTH) [72], thereby regulating pupal diapause in *Platysamia cecropia* [73], *Helicoverpa armigera* [74], and *Antherea pernyi* [75]. A study on the differential gene expression of diapause- and non-diapause-destined larval brains in bivoltine silkworms found that two genes (Cry18al and Kr-h1) related to ecdysone synthesis were upregulated in diapause-destined larval brains compared to the non-diapause-destined brains [76,77,78]. In addition, diapause-destined adults would lay non-diapause eggs after application of KK-42, an imidazole compound with anti-juvenile hormone and ecdysone effects, to diapause-destined bivoltine silkworms during the fifth instar [79]. The above findings suggest that ecdysone might be the endocrine signal responsible for transmitting diapause signals received during the BK phase, and this coincides with the fact that the growth rate of diapause-destined larvae is slower than that of non-diapause-destined larvae. Another enriched pathway, the FoxO signal pathway, also could affect the production of ecdysteroids [80,81] and ultimately regulate growth, a process that is basically the same in insects and nematodes [82]. Among those DEmiRs, two miRNAs (bmo-miR-2843-5p and novel_232) were predicated to regulate the ecdysone synthesis component of insect hormone biosynthesis, and the novel_232 was one of the top 10 DEmiRs in the HP groups. There was another miRNA in the top 10 DEmiRs, bmo-miR-3220, that was only expressed under the non-diapause-inducing temperature during the HP phase. The KEGG enrichment pathway analysis of DECT genes of the miRNA showed that this gene mainly participates in metabolism and signal molecules interaction. This result implies that bmo-miR-3220 might not only be involved in the lower developmental rates under low temperature but may also inhibit the interaction of diapause signal molecules, and eventually prevent the passing of diapause signals.

Surprisingly, there were nine and six bmo-miR-2733 family members significantly differentially expressed in different temperature-treated embryos during the BK and HP stages, respectively. After analyzing the predicted target genes of the bmo-miR-2733 family, we found that almost all of the differentially expressed family members could regulate the expression levels of two proteins, the translocation-associated membrane protein named TRAM and the two-pore domain potassium channel. There are reports that the TRAM protein is related to protein processing in the endoplasmic reticulum, and that it mainly participates in the process of protein synthesis [83]. Interestingly, two members of the two-pore domain potassium channel proteins, TWIK-2 and TREK-1, are densely concentrated in the dorsal root ganglion sensory nerve fibers and preoptic hypothalamic temperature receptor-dense area in mammals and are highly sensitive to temperature [84]. This suggests that the bmo-miR-2733 family might mediate the sensitivity of silkworm embryos to temperature signals by regulating the expression of a protein with a potassium channel domain, and then participate in the formation of a temperature-relevant phenotype, including the speed of embryonic development, through affecting protein processing in the endoplasmic reticulum.

## 5. Conclusions

Taken together, the results of our study demonstrate changes in the abundance of miRNAs, and there were differences in the GO and KEGG enrichment analyses of the target genes of DEmiRs in different temperature-treated embryos during the BK and HP phases. The finding that bmo-miR-6497-3p could regulate the expression of the core positive circadian clock gene cycle in vitro further indicates that some miRNAs can respond to temperature and may be involved in diapause induction by affecting the circadian clock during the BK phase. Our research provides further evidence that there is epigenetic regulation of the circadian clock mediated by miRNAs in the process of diapause induction under different temperatures. Moreover, our findings not only provide reference for the epigenetic modification in the diapause induction process of other species, but also contribute to the study of regulation by miRNA of other physiological processes.

## Data Availability

All of the raw sequence data were deposited in the NCBI Sequence Read Archive (SRA) under BioProject accession number PRJNA741626.

## Supplementary Materials

The following are available online at https://www.mdpi.com/article/10.3390/insects12080739/s1, Table S1: Major primer sequences in this study, Table S2: Information statistics on mapping with reference genome.
