# Supplementary material for: Comparative Transcriptome Analysis Reveals bmo-miR-6497-3p Regulate Circadian Clock Genes during the Embryonic Diapause Induction Process in Bivoltine Silkworm
Source: Insects. 2021 Aug 18;12(8):739. doi: 10.3390/insects12080739 (PMC8396838; doi:10.3390/insects12080739)
Supplement: Supplementary file 1 [file insects-12-00739-s001.zip › insects-1320994-supplementary.pdf]

Table S1. Major primer sequences in this study

Primer name	Primer sequence（5′-3′）
RNA5′Adapter	GTTCAGAGTTCTACAGTCCGACGATC
RNA3′Adapter	AGATCGGAAGAGCACACGTCT
bmo-miR-6497-3p-F for OE	CCACTAGTCCAGTGTGGTGGCCAAAAAGGTAATTCCATTTCC
bmo-miR-6497-3p-R for OE	ACTGTGCTGGATATCTGCAGTTTCCCAAGTCCCTCGCTC
bmo-miR-6497-3p-SL primer	CTCAACTGGTGTCGTGGAGTCGGCAATTCAGTTGAGAGACCCGG
bmo-miR-6497-3p-F	ACACTCCAGCTGGGGATGCGGCCGGTGCC
bmo-miR-6497-3p-R	TGGTGTCGTGGAGTCG
U6-F	CTCGCTTCGGCAGCACA
U6-R	AACGCTTCACGAATTTGCGT
Cycle-F	GCCAGTCAAGGAGGAGGT
Cycle-R	AGAGTTCGTGGTGGGAGC
Pdp-F	GAAGGAGAATATGGGCTTACGG
Pdp-R	GTGTTTGTGCTATGCGGGTG
SW22934-F	TTCGTACTGCTCTTCTCGT
SW22934-R	CAAAGTTGATAGCAATTCCCT
Cycle 3′UTR-F	GTCATGTGAAAACCTAGCCTTAGTG
Cycle 3′UTR-R	CATATCATCAGATGCTTTACCAGTTAG

†: the solid line indicates the sequence of homology arm primer for constructing overexpression vector, and the dotted line indicates the regular part in primer.

Table S2. Information statistics on mapping with reference genome

Sample	Total sRNA	Mapped sRNA	" + "Mapped sRNA	"-"Mapped sRNA
BK25	13172384 (100.00%)	11290246 (85.71%)	6815481 (51.74%)	4474765 (33.97%)
BK15	12211015 (100.00%)	10778240 (88.27%)	6660938 (54.55%)	4117302 (33.72%)
HP25	8595053 (100.00%)	7410664 (86.22%)	5103721 (59.38%)	2306943 (26.84%)
HP15	7543817 (100.00%)	6299620 (83.51%)	3800764 (50.38%)	2498856 (33.12%)
